# 

**Published:** 1848-01

**Authors:** 


### CONTENTS

OF NO. I.

### ORIGINAL COMMUNICATIONS.

### ESSAYS AND CASES.

ART. I.—Notes on Medical Matters and Medical Men in Paris. By DAVID W. YANDELL, M.D., of Louisville, Ky. ......... 1

ART. II.—A Case of Purpura Hemorrhagica. By SAMUEL B. ROBISON, M.D., of Rutherford county, Tennessee ........... 21

ART. III.—A Case of Poisoning by Tartar Emetic. By JOHN T. GLEAVES, M.D., of Green Hill, Tennessee ............ 23

### REVIEWS.

ART. IV.—A Treatise on the Physical Cause of the Death of Christ, and its Relation to the Principles and Practice of Christianity. By WM. STROUD, M.D. London. 1847 .... 26

ART. V.—The Dental Register of the West. Vol. I., No. I. Cincinnati. 1847 ........................................ 42

ART. VI.—New Elements of Operative Surgery. By ALF. A. L. M. VELPEAU, Professor of Surgical Clinique of the Faculty of Medicine of Paris, &c., &c. Carefully revised, entirely remodelled, and augmented with a Treatise on Minor Surgery. Illustrated by over 200 Engravings, incorporated with the text. Accompanied with an Atlas in quarto of twenty-two plates, representing the principal operative processes, surgical instruments, &c. First American, from the last Paris edition. Translated by P. S. TOWNSEND, late Physician to the Seamen's Retreat, Staten Island, New York. Augmented by the addition of several hunded pages of entirely new matter, comprising all the latest improvements and discoveries in Surgery, in America and Europe, up to the present time. Under the supervision of, and with notes and observations by, VALENTINE MOTT, M.D., Professor of the Operations of Surgery with Surgical and Pathological Anatomy, in the University of New York, &c., &c. In three volumes. Vol. I. pp. 914: 1845. Vol. II. pp. 1092: 1846. Vol. III. pp. 1162: 1847. New York: Samuel S. & William Wood.....50

SELECTIONS FROM AMERICAN AND FOREIGN JOURNALS.

Cases of Retained Placenta. By W. L. SUTTON., M.D., of Georgetown, Ky.....61

Fatal Hemorrhage from the Umbilical Cord three days after Birth.....70

Observations on the Cases of Yellow Fever received into the Marine Hospital, Charleston, from July, 1834, to November, 1838.....71

Cases of Periodical Asthma Checked by the Sulphate of Quinine. By Dr. J. C. CAIN, M.D.....74

Remarks on the Chemical Theory of Diet. By R. DICK, M.D.....75

Case of Poisoning with Arsenic.....76

On the Application of Bandages to the Lower Extremities in Uterine Hemorrhage.....78

Animal Heat as Affected by Phthisis. By SIR CHARLES SCUDAMORE.....80

A Case of Glossitis—Mal-treatment, nearly resulting in Death. By T. P. BICKNELL, M.D.....82

Sketches and Illustrations of Medical Quackery—Homœopathy.....83

On the Cutaneous Eruptions Induced by Various Medical Substances—Opium.....84

ORIGINAL INTELLIGENCE.

The Late Flood in the Ohio River.....85

The Liver not Extirpated.....87

San Leopoldo Sanitary Establishment, Havana.....88

Introductory Lectures.....88

Transactions of the Philadelphia College of Physicians.....90

Treatment of Idiots.....92

Epidemic Cholera.....92

Health of the City.....92

Poisoning by Arsenic.....92

